# Current Understanding of Pulmonary Fibrosis: Pathogenesis, Diagnosis, and Therapeutic Approaches

**DOI:** 10.1155/carj/3183241

**Published:** 2025-07-15

**Authors:** Dinglu Cui, Xiangguo Che, Rongxian An, Lei Li, Xinying Cui, Long Jiang, Jingchun Jin

**Affiliations:** ^1^Department of Rheumatology, Yanbian University Hospital, Yanji, China; ^2^Department of Biochemistry and Cell Biology, Korea Mouse Phenotyping Center, Cell and Matrix Research Institute, School of Medicine, Kyungpook National University, Daegu, Republic of Korea

**Keywords:** diagnosis, pathogenesis, pulmonary fibrosis, TCM prescription, traditional Chinese medicine, treatment

## Abstract

Pulmonary fibrosis (PF) is a terminal-stage lung change in interstitial lung disease. It is characterized by proliferation of fibroblasts and deposition of a large amount of extracellular matrix, accompanied by inflammatory damage and structural destruction, caused by various reasons. The prognosis of PF is poor, and the average survival time after diagnosis is 2.5–3.5 years. The pathogenesis of PF is not yet fully understood. Its main mechanisms are diverse and include damage to alveolar epithelial cells, aggregation and activation of inflammatory cells and chemokines, proliferation of fibroblasts, transformation of myofibroblasts, production and deposition of large amounts of collagen, autophagy, epithelial–mesenchymal transition (EMT), mitochondrial quality-control disorders, microRNA, and circular RNA. The diagnosis of PF is mainly based on the comprehensive evaluation of clinical manifestations, imaging characteristics, and histopathological examination. Medical and family history to determine all potential causes of PF. For PF of unknown etiology, one can refer to the Official Clinical Practice Guideline of idiopathic pulmonary fibrosis (IPF) for definitive diagnosis. In terms of treatment, modern medications such as pirfenidone and nintedanib can inhibit the progression of PF to some extent and improve lung function. However, there is no drug that can significantly improve PF, except for lung transplantation. In addition, many patients are forced to stop taking medication due to adverse reactions in clinical practice. Therefore, to better control the progression of disease, some new drugs have been developed based on the pathogenesis of PF. However, there is still controversy over their efficacy and widespread clinical application in PF, and the evidence is limited. The results of in vitro and in vivo experiments, as well as randomized clinical trials, indicate that traditional Chinese medicine (TCM) can improve PF by intervening in multiple pathways and targets. This study combines the pathogenesis and diagnosis of PF, focusing on the intervention mechanism and targets of TCM in the treatment of PF, so as to provide more options for clinical treatment and provide scientific basis for a new approach to better management of PF.

## 1. Introduction

Pulmonary fibrosis (PF) is a terminal interstitial lung disease (ILD) characterized by fibroblast proliferation and deposition of large amounts of extracellular matrix (ECM) along with inflammatory damage and tissue structure destruction caused by various factors (such as occupational and environmental factors, radiation therapy, drugs, and connective tissue diseases) [1]. The growing incidence of PF [2] has made this disease an urgent health problem of global concern. The prognosis of PF is poor, with most patients showing a gradual decline in pulmonary function and eventual intractable respiratory failure. Specifically, the average survival time after diagnosis is 2.5–3.5 years [3, 4].

The pathogenesis of PF remains unclear. The structural changes caused by PF are irreversible, and no existing therapeutic agent has proven to effectively stop the progression of the disease. Although a growing number of new drugs have been developed to better control the disease, further confirmation of their effectiveness is required. In addition, traditional Chinese medicine (TCM) has shown to have anti-inflammatory, antioxidative, and antifibrotic effects in the treatment of PF [5]. TCM treatment can effectively improve the overall pathological state of patients and their quality of life while causing limited adverse reactions.

This review discusses the pathogenesis, diagnosis, and treatment of PF in order to provide a theoretical basis for clinical diagnosis and treatment of PF and serve as a reference and inspiration for further research on PF treatment.

## 2. Pathogenesis

**Figure 1:**
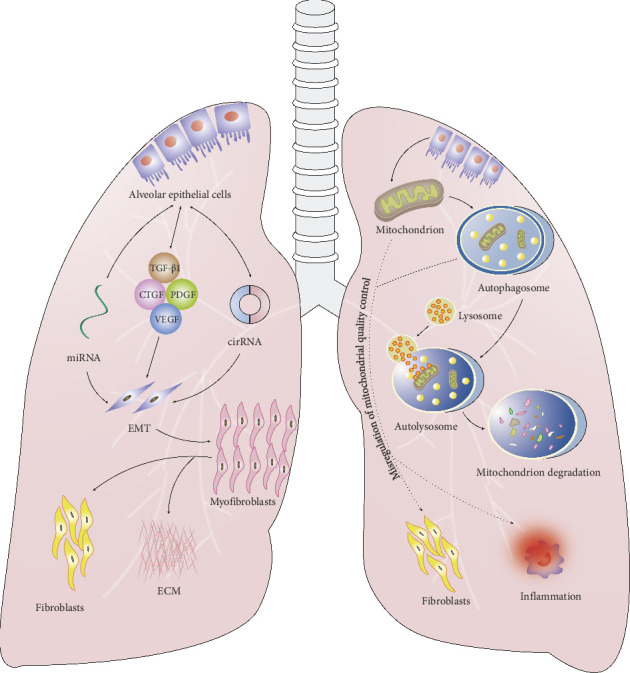
Main mechanisms of PF include damage to alveolar epithelial cells, aggregation and activation of inflammatory cells and chemokines, transformation of myofibroblasts, deposition of a large amount of ECM, autophagy, EMT, mitochondrial quality-control disorders, microRNA (miRNA), and circular RNA (cirRNA). PF: pulmonary fibrosis; EMT: epithelial–mesenchymal transition; ECM: extracellular matrix; TGF-β1: transforming growth factor-β1; CTGF: connective tissue growth factor; PDGF: platelet-derived growth factor; VEGF: vascular endothelial growth factor.

The mechanisms underlying PF include damage to alveolar epithelial cells (AECs), the effects of profibrotic factors, fibroblast proliferation, myofibroblast transformation, autophagy, epithelial–mesenchymal transition (EMT), misregulation of mitochondrial quality control, and the activities of microRNAs (miRNAs) and circular RNAs (circRNAs) (Figure 1).

### 2.1. AECs

AECs form a complete epithelial layer on the alveolar surface. They are classified into Types I and II based on their structure and function. Type I AECs cover 95% of the alveolar surface and are the primary medium for gas exchange. Type II AECs are mainly distributed between Type I AECs and their junctions with the adjacent alveolar septum and cover approximately 5% of the alveolar surface. When the lung parenchyma is injured, Type II AECs rapidly proliferate and differentiate into Type I AECs to restore normal lung function [6, 7] and can also replenish their own number by mitosis; therefore, they are considered the stem cells of the alveolar epithelium [8, 9]. Type II AECs are the main cells that synthesize and secrete alveolar surfactant and its related proteins [10]. Abnormalities in the functional state of Type II AECs are considered to be related to a variety of lung injuries and diseases [10].

At present, impaired homeostasis of AECs is generally considered to be the main driving factor in the pathogenesis of PF, and abnormalities in Type II AECs play an important role in this process [11]. In comparison with healthy lungs, the lungs of patients with PF display higher levels of apoptosis, senescence, abnormal differentiation, and impaired regeneration of Type II AECs [11]. The mechanisms involved in these changes include cell senescence, telomere shortening, mitochondrial dysfunction, endoplasmic reticulum (ER) stress, autophagy, matrix metalloproteinase (MMP) expression, EMT, and the activity of Wnt signaling pathways, which are present in almost all lungs with idiopathic PF (IPF) [12–14].

In patients with IPF and bleomycin-induced PF mouse models, Type II AECs express senescence-associated β-galactosidase (SA-β-gal) and also show increased expression of secretory phenotype (SASP) markers such as secretory pyrophosphoprotein (SPP) 1 and MMP2. The antiaging drugs dasatinib and quercetin have shown to significantly inhibit cell senescence and fibrosis [15].

Type II AECs of mice missing the gene-encoding glucose regulatory protein 78 (GRP78)—a key ER stress regulatory protein—show ER stress injury, apoptosis, aging, and decreased differentiation ability, which is accompanied by abnormal activation of transforming growth factor (TGF)-β1 signaling. These results indicate that abnormal ER stress can lead to dysfunction of the alveolar epithelium and that ER stress may be a potential mechanism that links aging with IPF. Therefore, regulating ER stress may be a promising treatment target for PF [16].

Type II AECs with positive Axin2 expression can receive Wnt secretion signals from neighboring fibroblasts and maintain their stem cell characteristics, thereby promoting the activity and proliferation of Type II AECs. When lung injury occurs, Type II AECs secrete Wnt signals, temporarily expand stem cell reserves, and differentiate into Type I AECs to repair the injury [14].

### 2.2. Profibrotic Factors

#### 2.2.1. TGF-β1

TGF-β1 is an essential factor for lung organogenesis and homeostasis and is involved in the development of many respiratory diseases, including PF, emphysema, and lung cancer. TGF-β1 is currently recognized as an important initiator of PF, playing a central role in disease regulation [17] potentially through the activation of a variety of downstream Smad proteins [18]. The TGF-β1 signaling pathway is widely involved in the occurrence and development of PF, and its effects are closely related to the fact that TGF-β1 can induce cell differentiation, migration, invasion, or proliferative changes [19].

An increasing number of studies have shown that TGF-β1 plays an important role in the development of PF. The main mechanisms through which TGF-β1 can induce PF are as follows: ① TGF-β1 can activate AECs and promote alveolar epithelial injury and apoptosis; ② TGF-β1 can mediate the process of EMT by activating the Smad pathway and inhibit the growth and repair of AECs; ③ TGF-β1 can directly stimulate myofibroblasts to express ECM genes and enhance the secretion of ECM by myofibroblasts; ④ TGF-β1 can also inhibit the production of MMPs, promote the production of tissue inhibitors of MMPs (TIMPs), and eventually inhibit the degradation of ECM in lung tissue, resulting in the deposition of large amounts of ECM; ⑤ TGF-β1 can cooperate with cytokines such as connective tissue growth factor (CTGF), platelet-derived growth factor (PDGF), and vascular endothelial growth factor (VEGF) to further accelerate the progression of PF [20, 21].

#### 2.2.2. CTGF

CTGF is a cytokine that plays a key role in a variety of fibroproliferative diseases [22]. It is regarded as a downstream response element of TGF-β1 that can promote fibroblast proliferation and participate in ECM production, accumulation, and angiogenesis [22]. Under pathological conditions, CTGF is overexpressed in fibrotic lesions of the lungs, kidneys, liver, skin, cardiovascular system, gastrointestinal system, eyes, and other major organs [23].

As a primary mediator of TGF-β1-induced PF, CTGF has a strong regulatory effect on the differentiation and proliferation of lung fibroblasts. Vanstapel et al. reported high expression of CTGF in the fibrotic regions of lungs with restrictive allograft syndrome (RAS) [24]. Other studies have shown that CTGF is upregulated in patients with IPF as well as in profibrotic mediators and profibrogenic environments and may help maintain abnormal responses of AECs, fibroblasts, and alveolar macrophages during the course of IPF [25]. CTGF also plays direct and indirect roles in accelerating aging and mitochondrial dysfunction [26, 27].

#### 2.2.3. PDGF

PDGF is a low molecular weight cytokine stored in platelet alpha granules. The PDGF protein family consists of four ligands, namely, A, B, C, and D, which are mitogens and chemotactic agents of mesenchyme-derived cells [28] and regulate cell proliferation, migration, and survival, as well as ECM synthesis and secretion [29]. During injury, epithelial cells, endothelial cells, macrophages, and immune cells secrete PDGF to promote the proliferation and migration of their own cell populations [29].

PDGF is highly expressed in sulfur mustard (SM)– and bleomycin-induced PF models, significantly more than in the model group, and PDGF content has shown to decrease after treatment with pirfenidone and simvastatin [30–32].

PDGFs have also shown to be expressed in a bleomycin-induced PF mouse model, with the most significant changes observed in PDGF-C and PDGF-D expression [33]. While normal lung tissue shows almost no expression of PDGF-C, its expression is significantly increased in fibrotic lung tissue. In contrast, PDGF-D is constitutively expressed in normal lung tissue, whereas it is downregulated in bleomycin-induced PF models [34, 35]. Thus, PDGF-C promotes PF development, whereas PDGF-D may be negatively associated with PF development.

#### 2.2.4. VEGF

VEGF is a highly specific growth factor that can promote vascular permeability, ECM degeneration, vascular endothelial cell migration and proliferation, and angiogenesis. It plays a crucial role in the dynamic balance of diseases and regulation of angiogenesis. The growing body of research on the relationship between VEGF and PF has shown that VEGF can promote the progression of PF by increasing the expression of MMPs and participating in the PI3K/Akt signaling pathway, the JAK2/STAT3 signaling pathway, macrophage inflammation, hypoxia regulation, and multiple TGF-β1 pathways [36–41]. Therefore, reducing VEGF expression may help inhibit or treat IPF. However, some researchers have also pointed out that VEGF exerts protective effects against PF [42, 43]. Experimental studies have shown that low VEGF expression is associated with poor clinical outcomes and that patients with IPF and higher circulating VEGF levels have better lung function and a more benign clinical phenotype [42]. Thus, VEGF may promote vascular proliferation, which may contribute to the regeneration of lost alveolar walls during fibrosis and the repair of fibrosis.

Therefore, the relationship between VEGF and PF remains inconclusive, and further studies are needed to clarify it.

### 2.3. Fibroblasts and Myofibroblasts

#### 2.3.1. Fibroblasts

The formation, activation, and aggregation of fibroblasts are the most direct reasons for the occurrence and development of IPF [44, 45]. Under specific pathological conditions, a large number of fibroblasts are derived from the following three sources: differentiation of pulmonary mesenchymal stem cells (MSCs) [46–49], transformation of AECs and EMT [50, 51], and migration of fibrotic cells from circulating blood [52, 53]. These sources and lung-resident fibroblasts gather at the injury site and proliferate and differentiate into myofibroblasts with stronger contractility. Myofibroblasts secrete large amounts of ECM, including collagen and fibrin, which thickens the alveolar wall, leading to fibrous scar formation, alveolar structural disorder, and loss of function, eventually leading to PF [54, 55].

The Wnt/β-catenin signaling pathway plays an important role in the differentiation of pulmonary MSCs into fibroblasts, and fibroblasts into myofibroblasts. Thus, blocking the Wnt/β-catenin signaling pathway can effectively inhibit the differentiation of pulmonary MSCs into fibroblasts, and also inhibit the transformation of fibroblasts into myofibroblasts, thereby inhibiting the progression of PF [56, 57].

#### 2.3.2. Myofibroblasts

Regardless of the cause of PF, myofibroblasts play a key role in the chronic inflammatory response and progression of fibrosis [58, 59]. Pulmonary myofibroblasts have a strong ability to synthesize ECM, and ECM deposition leads to remodeling of the lung structure and loss of alveolar function [60].

In addition to transformation from lung-resident fibroblasts, pulmonary myofibroblasts may also be transformed from epithelial cells, endothelial cells, adipose fibroblasts, circulating fibroblasts derived from the bone marrow, and even macrophages. All of these cells can be activated by TGF-β1 and transformed into pulmonary myofibroblasts. Pirfenidone and nintedanib, the main drugs currently used for the treatment of PF, play antifibrotic roles by inhibiting the transformation of PFs into pulmonary myofibroblasts [61].

Numerous studies have shown that the main mechanisms underlying pulmonary myofibroblast transformation are as follows: ① Mechanical transduction: Mechanical interactions between pulmonary myofibroblasts and the hardened ECM exacerbate the development of PF [62–64]. ② Metabolic disorders: Increased glycolysis, increased glutamine decomposition, and decreased lipid production may be important drivers of lung fibroblast activation [65, 66]. ③ Oxidative stress: A persistently high concentration of reactive oxygen species (ROS) can cause oxidative stress in the body, activating the body's repair and defense mechanisms, damaging lung tissue, and accelerating the progression of PF [67]. Thus, activation of pulmonary myofibroblasts can be prevented by regulating oxidative stress [68]. ④ Ubiquitin: E3 ubiquitin ligase and deubiquitination enzymes regulate the ubiquitin degradation of PF-related proteins and affect the transformation process of pulmonary myofibroblasts [69, 70]. ⑤ Cellular aging: In patients with PF, aging lung fibroblasts may develop apoptosis resistance that is difficult to clear, and continuous secretion of age-related secretory phenotypic factors may promote the transformation of pulmonary myofibroblasts, thereby driving the process of PF [71, 72].

### 2.4. EMT

EMT is the process of dedifferentiation of AECs into mesenchymal cells that secrete ECM, which perpetuates fibrotic lesions and increases the stiffness of the lung tissue structure [73]. During EMT, AECs lose their apical and basal polarities and exhibit reduced intercellular adhesion. They also start expressing mesenchymal markers such as α-smooth muscle actin (α-SMA), N-cadherin, vimentin, and EMT-related transcription factors, and undergo cytoskeletal reorganization [74, 75].

The histopathological features of PF include progressive and unevenly distributed scarring at the base and sides of the lungs [76], and the characteristic hallmark of these scarring areas is the presence of clusters of fibroblasts (fibroblast foci). The origin of fibroblasts and myofibroblasts in PF fibroblast clusters has been a longstanding topic of debate. Most researchers believe that they are mainly derived from resident tissue fibroblasts, bone marrow–derived progenitor cells (the so-called fibroblasts), and Type II AECs that undergo EMT. The secretion of ECM by fibroblasts and myofibroblasts derived from EMT leads to PF progression, which plays a key role in the pathophysiological process of PF [77].

During the activation of EMT signals, the tissue microenvironment determines whether cells undergo EMT [78]. In chronic diseases, the microenvironment at the injury site is characterized by hypoxia, chronic inflammation, oxidative stress, disordered cytokine secretion, and increased ECM stiffness, all of which serve as potential triggers of EMT, thereby activating the EMT signaling pathway. The signal transduction pathways that regulate EMT mainly include the TGF-β-Smad, Wnt/β-catenin, Hippo, Notch, and NF-κB pathways [79–81]. Therefore, by elucidating EMT-related signaling pathways, identification of drugs that target EMT may facilitate the treatment of PF.

### 2.5. Autophagy

Autophagy has been widely observed in eukaryotic cells and organisms. This process involves the degradation of old intracellular proteins and organelles through the lysosomal pathway and recycling of small molecules, such as amino acids and nucleic acids, to meet the cells' own metabolic needs and facilitate the renewal of specific organelles. Moderate autophagy maintains homeostasis, while excessive autophagy induces cell damage [82]. Autophagy is regulated by various autophagy-related genes (ATC), proteins (LC3, p62, and ULK1), and multiple signaling pathways. The mammalian target of rapamycin (mTOR) is a key factor in regulating autophagy and participates in multiple autophagy signaling pathways related to PF. The PI3K/Akt/mTOR and MAPK/mTOR pathways negatively regulate autophagy, while the AMPK/mTOR pathway positively regulates autophagy [83]. Among these, the PI3K/Akt/mTOR signaling pathway has been extensively studied.

A growing body of the literature has shown that autophagy dysfunction plays an important role in the occurrence and development of PF. Autophagy dysfunction mainly promotes PF through pathways such as inflammatory responses, ER stress, oxidative stress, EMT, ECM deposition, cell apoptosis, cell aging, angiogenesis, and regulation of AEC function [84–90]. Thus, autophagy is an important component of the pathogenesis of PF.

### 2.6. Misregulation of Mitochondrial Quality Control

Mitochondria undergo quality control, which involves changing their shape and size through fusion/division, enveloping damaged mitochondria through mitochondrial autophagy, transferring them to lysosomes for clearance, supplementing the mitochondrial pool through the biosynthesis of new mitochondria, and reducing the oxidative damage to cells caused by ROS through the mitochondrial antioxidant system.

Mitochondrial quality control has recently shown to play an important role in PF [91]. In PF, dysregulation of mitochondrial quality control leads to mitochondrial dysfunction, increased ROS production, induction of cell apoptosis, enhanced mitochondrial fusion, and reduced mitochondrial autophagy and biosynthesis. Mitochondrial dynamics and PF are closely related, and changes in mitochondrial dynamics may affect the cellular energy supply and signal transduction by affecting mitochondrial ROS (mtROS) generation, mitochondrial DNA (mtDNA) distribution, intracellular calcium homeostasis, and cell apoptosis, thereby promoting the occurrence and development of PF [92, 93]. In addition, excessive mtROS production and decreased activity of protease Complexes I and IV have been reported to be associated with mitochondrial dysfunction in Type II AECs in a mouse model of PF [94, 95].

Mitochondrial biosynthesis is a biological process that regulates the formation of newborn mitochondria and mainly involves the replication of mtDNA, synthesis of mtDNA-encoded proteins, and coordination of mitochondrial dynamics. The key regulatory molecule in mitochondrial biosynthesis is peroxisome proliferator-activated receptor-γ coactivator (PGC)-1α. Decreased mitochondrial biosynthesis is associated with the pathogenesis of PF. In patients with PF and bleomycin-induced PF models, the expression level of PGC-1α is reduced. In addition, the degree of PF in PGC-1α gene-knockout mice has shown to worsen after induction by bleomycin [96].

Evidence of mitochondrial dysfunction leading to the fibrotic response is particularly evident in PF; however, the relevant mechanisms linking mitochondrial quality control and abnormal repair in PF require further exploration.

### 2.7. miRNAs

miRNAs are a type of functional noncoding single-stranded small RNA in eukaryotes with a length of approximately 20–24 nucleotides. miRNAs are produced from pre-miRNAs via Dicer enzyme cleavage. Pre-miRNAs are composed of approximately 70–90 bases with a hairpin structure. miRNAs play regulatory roles in various diseases such as psoriasis, cancer, and fibrotic diseases by inhibiting translation processes or degrading target mRNA [97, 98].

Research on miRNAs has become increasingly popular. As key small RNA molecules that regulate gene expression, miRNAs play important roles in the occurrence and development of PF [99] and participate in lung epithelial repair, EMT, fibroblast activation, myofibroblast differentiation, macrophage polarization, aging of AECs, and collagen generation during PF.

The miRNAs that are upregulated during PF are primarily miR-21 and miR-155 [100, 101]. The introduction of miR-21 antisense probes or knockout of miR-21 genes in experimental mice has shown to alleviate PF, while the introduction of miR-21 precursor substances has shown to exacerbate PF, indicating that miR-21 has a fibrogenic effect [100]. miR-21 mimetics and miR-21 inhibitors have also been used to regulate lung fibroblasts in vitro: The former promotes the proliferation of lung fibroblasts and collagen synthesis, whereas the latter has the opposite effect [102], indicating that miR-21 is an important regulatory factor in the pathogenesis of PF and may be a potential target for treating patients with PF.

Kurowska-Stolarska et al. demonstrated that knocking out the miR-155 gene promoted collagen deposition, Collagen 1 and 3 mRNA expression, TGF-β1 production, and the activation of alternatively activated macrophages, thereby aggravating the occurrence of PF in mice. However, when liver X receptor α—the target factor of miR-155—was inhibited, PF was delayed [101].

The main miRNAs downregulated in the pathogenesis of PF are miR-26α, miR-29, miR-149, miR-200 family, and miR-101 [103–109]. Inhibition of miR-26α causes lung epithelial cells to transform into myofibroblasts and aggravates PF; overexpression of miR-26α can reduce the production of EMT and delay PF [103]. In addition, when PF occurs, downregulation of miR-29 expression increases the expression of TGF-β and CTGF and the phosphorylation of Smad3 and weakens the antifibrotic effects of the PI3K/AKT pathway and the Wnt/β-catenin signaling pathway, thereby promoting PF [104].

miRNAs can also regulate autophagy. miR-101 inhibits the activation of autophagy and reduces cell death by downregulating the expression of its target genes STMN1, RAB5A, and ATG4D [105]. miR-101 has also shown to inhibit NFATc2 signal transduction by targeting Frizzled receptor 4 × 6, inhibiting WNT5a-induced PF proliferation, and inhibiting TGF-β–induced PF activation by targeting TGF-β1 receptor inhibition of SMAD2/3 signal transduction [106].

The human serum contains abundant amounts of stable miRNAs that are closely related to the occurrence and development of PF. The expression profile of miRNAs also changes with the pathological status and disease progression. Specifically, a study on serum miRNAs in patients with IPF showed that the levels of miR-21 and miR-155 were significantly increased in the serum of patients with PF, and the expression level of miR-21 was proportional to the degree of PF [110]. The downregulation of miR-101-3p was the most significant. Therefore, serum miRNAs may be candidates for early diagnostic biomarkers of PF; however, the levels of miRNAs in serum are very low. In this context, further exploration is needed regarding their secretion mode, protective mechanisms for maintaining stability in the blood, and the impact of coagulation processes on miRNAs [111].

### 2.8. circRNAs

circRNAs are noncoding RNAs with a covalently closed loop structure formed by reverse cleavage of precursor mRNAs. Unlike linear mRNAs, circRNAs are not affected by RNA exonucleases and are not easily degraded; therefore, they are more stable [112]. circRNAs have shown to play an important role in various biological functions such as cell proliferation, migration, invasion, and pluripotency [113]. circRNAs can serve as competitive endogenous RNA or protein-coding RNA or interact with RNA-binding proteins (RBPs) and widely participate in the physiological and pathological processes of diabetes, nervous system diseases, fibrosis, and other diseases [114].

Several studies have shown that circRNAs are involved in the pathogenesis of PF. The regulatory roles of circRNAs in PF mainly include the following aspects: blocking EMT; inhibiting the activity and proliferation of fibroblasts; blocking the transformation of fibroblasts into myofibroblasts; regulating macrophages; and regulating the TGF-β1 signaling pathway, ER stress, and other aspects [115–119].

Additionally, circRNAs may serve as potential therapeutic targets and biomarkers for the diagnosis of PF. Li et al. identified 67 severely dysregulated circRNAs in the plasma of patients with PF using a circRNA expression profile chip: 38 were upregulated and 29 were downregulated [120]. To date, many circRNAs have been found to be associated with PF; however, the link between circRNAs and PF remains to be elucidated. In the future, circRNAs may be identified as important participants in gene regulation, and exploring their role in the diagnosis of PF and gene therapy will be of great significance.

## 3. Diagnosis

The diagnosis of PF is mainly based on a comprehensive evaluation of clinical manifestations, imaging characteristics, and histopathological findings. Among these methods, histopathological assessments are considered the gold standard. Several substances and conditions can cause PF. However, in most cases, the cause of the disease cannot be identified. PF with no known cause is known as IPF. IPF is characterized by usual interstitial pneumonia (UIP) [121].

### 3.1. Symptoms

The onset of PF is usually asymptomatic, and its main clinical manifestations are dyspnea, progressive exacerbation, and dry cough. Systemic symptoms are not obvious; fatigue and weight loss may occur but fever is rare. In approximately half of the patients, clubbed fingers develop, and in 90% of the patients, small velcro rales at the end of inspiration can be heard at the base of both lungs.

### 3.2. Imaging Characteristics

#### 3.2.1. Chest Radiography

Chest radiographs of patients with PF show obvious reticular or reticular nodules in the outer zones of both lungs and the subpleural and basal areas, which are accompanied by honeycomb-like changes. Although this finding has low sensitivity and specificity for diagnosing PF, it can be used as a preliminary screening test for lung disease [122].

#### 3.2.2. High-Resolution Computed Tomography (HRCT)

HRCT is an important tool for diagnosing PF. The 2022 ATS/ERS/JRS/ALAT Guidelines defined the following imaging findings for evaluation of PF [121]: ① UIP pattern: honeycombing with or without traction bronchiectasis/bronchiolectasis, presence of irregular thickening of the interlobular septa usually superimposed with a reticular pattern, mild ground-glass opacity (GGO), and possible pulmonary ossification; ② probable UIP pattern: reticular pattern with traction bronchiectasis/bronchiolectasis, possible mild GGO, and absence of subpleural sparing; and ③ indeterminate for UIP: HRCT features of PF that do not indicate any specific etiology.

#### 3.2.3. Histopathology

For cases showing atypical UIP changes on HRCT, histopathological examination of the lungs can be performed when the diagnosis is unclear. The pathological diagnostic criteria for grading of UIP are as follows [123]: ① Typical UIP: evidence of marked fibrosis/architectural distortion with or without honeycombing in a predominantly subpleural/paraseptal distribution, presence of patchy involvement of the lung parenchyma by fibrosis, presence of fibroblast foci, and absence of features against a diagnosis of UIP that would suggest an alternative diagnosis. ② Probable UIP: evidence of marked fibrosis/architectural distortion with or without honeycombing, absence of patchy involvement or fibroblastic foci, but not both and absence of features against a diagnosis of UIP that would suggest an alternative diagnosis. ③ Possible UIP: patchy or diffuse involvement of the lung parenchyma by fibrosis with or without interstitial inflammation, absence of other criteria for UIP, and absence of features against a diagnosis of UIP that would suggest an alternative diagnosis. ④ Not UIP: hyaline membranes, organizing pneumonia, granulomas, marked interstitial inflammatory cell infiltrate away from honeycombing, predominant airway-centered changes, or other features suggestive of an alternative diagnosis.

Common methods of lung biopsy include transbronchial lung cryobiopsy (TBLC) and surgical lung biopsy (SLB). As pointed out by the ATS/ERS/JRS/ALAT Clinical Practice Guideline [121], TBLC is a reasonable alternative to SLB, especially in patients at a high risk of severe complications. However, TBLC cannot easily recognize subpleural pathological changes, and the possibility of sampling errors makes the application of UIP histopathological standards to TBLC specimens challenging. In comparison with SLB, considering the limited sampling of the subpleural lung parenchyma, TBLC is more likely to exhibit a possible UIP pattern than the typical UIP pattern in most cases [124]. Nevertheless, a combination of patchy fibrosis, fibroblast foci, and the absence of features suggesting an alternative diagnosis is usually sufficient for establishing a probable UIP pattern in TBLC [125]. In multidisciplinary discussion related to UIP and possible UIP types, the diagnostic consistency of SLB and TBLC in patients with IPF has shown to be similar [124].

In addition to the abovementioned diagnostic evidence, the diagnosis of PF should also be combined with medical and family history inquiries to determine all potential causes of PF, including dust, gas, or chemical exposure history, medication history (e.g., amiodarone, nitrofurantoin, and methotrexate), and history of underlying diseases (mainly connective tissue diseases such as systemic lupus erythematosus, rheumatoid arthritis, systemic sclerosis, idiopathic inflammatory myopathy, and ANCA-associated vasculitis).

Furthermore, a growing number of PF-related biomarkers, including genomic, proteomic, cellular, and microbiome biomarkers, have been identified [1] and can be used to assess PF type and risk of disease progression [126–128]. These biomarkers can distinguish patients with PF from healthy individuals, but they have limited ability to identify the types of PF, such as IPF or other fibrotic ILDs. Enzymes that influence ECM remodeling and have potential diagnostic utility as markers of disease activity and risk of progression include MMP-1 and MMP-7, as well as circulating proteins degraded by MMPs or circulating proinflammatory peptides [127, 128]. However, the use of a combination of two or three biomarkers to differentiate IPF from various CTD-ILDs lacks sensitivity and specificity. Nonetheless, peripheral blood sampling and the measurement of specific biomarkers may ultimately prove useful in distinguishing IPF from other fibrotic ILDs, and more studies on this topic are required.

## 4. Treatment

Modern medicine currently offers no substantially effective therapeutic options for PF, except lung transplantation. However, widespread clinical use of lung transplantation is difficult owing to the scarcity of donor organs and the high cost of surgery.

### 4.1. Modern Medical Treatment

The existing body of the literature in modern medicine does not identify any medication that can significantly improve PF. Traditional clinical treatment with corticosteroids and immunosuppressants can inhibit the progression of PF to a certain extent, but the curative effect of this treatment approach is poor. Although this treatment can improve the survival rate, its adverse effects are relatively substantial. Therefore, treatment regimens based on glucocorticoids, glucocorticoids + immunosuppressants, and glucocorticoids + immunosuppressants + N-acetylcysteine (NAC), and interferon are not recommended. The results of several recent randomized controlled trials have suggested that both pirfenidone and nintedanib can delay the progression of PF and help improve lung function, and their clinical application is relatively safe [129, 130]. These two drugs are the only anti-PF drugs approved by the US Food and Drug Administration (FDA).

#### 4.1.1. Pirfenidone

Pirfenidone is a multifunctional pyridine compound with antifibrotic, anti-inflammatory, and antioxidant properties. It can directly affect the expression of TGF-β1 by disrupting the interaction between the GLI-2 protein and the hedgehog signaling pathway; it can reduce the proliferation of fibroblasts by reducing the phosphorylation of the downstream intracellular proteins Smad3, p38, and Akt of the TGF-β signaling pathway [131], and it can also directly inhibit collagen secretion by lung fibroblasts and hinder the formation of outer collagen fibers. Through these effects, pirfenidone can safely stop further progression of fibrotic lesions, reduce excessive fibrotic lesions or scar tissue, and prevent the occurrence of fibrotic lesions.

In addition, pirfenidone has shown to inhibit γ-interferon to improve the Th1/Th2 balance and inhibit the proinflammatory cytokines tumor necrosis factor (TNF)-α and interleukin (IL)-6. Pirfenidone also exerts anti-inflammatory and antioxidant effects by promoting the production of the anti-inflammatory cytokine IL-10 and alleviating oxidative stress [132].

The unique antifibrotic and anti-inflammatory mechanisms of pirfenidone not only delay the progression of chronic fibrosis but also effectively alleviate the acute inflammation in PF by inhibiting inflammatory factors. A real-world study exploring the effects of antifibrotic therapy on the progression and survival of PF showed that patients treated with pirfenidone had a survival time of 3.5 years, which was significantly longer than that of the control group (2.5 years) [133]. The occurrence of adverse reactions should be monitored during pirfenidone treatment. The most common symptoms are gastrointestinal and skin symptoms, but they are mostly mild-to-moderate and reversible [134]. A meta-analysis of five randomized controlled trials assessing the use of pirfenidone for the treatment of PF showed that the incidence of adverse reactions in patients receiving pirfenidone was comparable to that of a placebo group [135]. Therefore, pirfenidone is a relatively safe and reliable treatment option for PF.

#### 4.1.2. Nintedanib

Nintedanib is a multitarget, small-molecule tyrosine kinase inhibitor with antifibrotic and anti-inflammatory activities. It can inhibit various receptor tyrosine kinases (RTKs), including PDGF receptors α and β (PDGFRα and PDGFRβ), fibroblast growth factor receptors 1–3 (FGFR1–3), VEGF receptors 1–3 (VEGFR1–3), and Fms-like tyrosine kinase 3 (FLT3), among which FGFR, PDGFR, and VEGFR are associated with the pathogenesis of PF [136]. Nintedanib competitively binds to adenosine triphosphate (ATP)–binding sites on these intracellular receptor kinase domains, thereby blocking intracellular signal transduction and inhibiting fibroblast proliferation, migration, and transformation. In addition, nintedanib can inhibit some non-RTKs (nRTKs), such as Lck, Lyn, and Src kinases, which can reduce the absolute value of the decline in forced vital capacity (FVC) in patients with PF and alleviate the progression of the disease to a certain extent [137]. The most common adverse reactions associated with nintedanib are diarrhea, nausea, vomiting, abdominal pain, and loss of appetite. Most of these symptoms are not serious, and less than 5% of patients discontinue the drug because of adverse reactions [137].

#### 4.1.3. NAC

NAC exerts direct antioxidant effects through a free thiol group (nucleophilic SH), which can directly interact with electrophilic oxidation groups while also protecting α-1. NAC also exerts a strong mucolytic effect by decomposing mucin complexes and nucleic acids, reducing the thickness of purulent components and mucus and mucus secretions in the sputum. NAC has shown antifibrotic and anti-inflammatory effects in patients with PF and can improve PF by promoting immune responses and inhibiting the EMT process through the VWF/p38MAPK axis [138]. NAC also shows potential antifibrotic effects by reducing the levels of hydroxyproline and TGF-β, and anti-inflammatory effects by reducing IL-17 levels [139]. NAC monotherapy cannot reduce the frequency of acute exacerbations and mortality of PF in general but can improve the sustained decline in FVC in some early-stage patients [140]. In addition, NAC reduces sputum production in patients with PF, and it is safe and can be used for a long time [140]. However, the literature contains conflicting evidence for combination therapy with pirfenidone and NAC: while a case-control clinical study suggested that NAC combined with pirfenidone was superior to pirfenidone alone in the treatment of intermediate- to late-stage PF [141], a recent randomized trial showed that inhaling NAC in combination with pirfenidone is not as effective as using pirfenidone alone for the treatment of PF [142]. Therefore, additional large-scale studies are needed to validate this combination therapeutic strategy.

#### 4.1.4. Antacids

Acid suppressants can reduce the risk of gastroesophageal reflux–related lung injury; therefore, the 2015 guidelines recommended the use of antacids in all patients with PF to delay the decline in lung function [143]. However, the latest guidelines suggest that routine use of antacids is not necessary in the treatment of patients with PF, with evidence showing that the use of antacids has no significant impact on the progression, lung function, adverse reactions, or mortality of PF [121].

In addition to the therapeutic agents mentioned above, protein therapeutic agents such as the antioxidant DHNNPQIR-NH2 (DR8), protein tyrosine phosphatase N13 (PTPN13) inhibitors, and MSCs have shown therapeutic potential for PF in multiple studies [143–145]. According to these reports, an oxidative/antioxidant imbalance is a key mechanism leading to PF, and DR8 has strong antioxidant activity. DR8 can prevent and treat PF by reducing oxidative damage and inhibiting the TGF-β/MAPK pathway [144]. Another study showed that PTPN13 is expressed in PF lung fibroblasts, promoting their resistance to Fas-induced cell apoptosis and PF in mice [145]. Therefore, interference with the expression or function of PTPN13 may represent a new strategy for delaying PF. Research has suggested that mDASCs could ameliorate bleomycin-induced PF by promoting lung regeneration and inhibiting lung fibrogenesis [146]. In addition, bone marrow mesenchymal stem cell (BMSC) transplantation has shown to inhibit EMT to alleviate silica-induced PF in rats and potentially show antifibrotic effects by attenuating Wnt/β-catenin signaling [147]. Stem cell therapy for PF also has enormous potential.

#### 4.1.5. Gene Therapy

In recent years, gene therapy, an emerging treatment strategy, has gradually garnered widespread attention and shown promising therapeutic prospects. It aims to prevent the occurrence and progression of diseases by regulating, repairing, or replacing abnormal genes in patients. A typical gene therapy approach involves using gene transduction technology to deliver antifibrotic genes to the patient's lung tissue, thereby blocking or reversing the disease progression of PF [148]. Research has found that employing CRISPR/Cas9 gene editing technology, Ad-CRISPR-T TGF-β1 can improve the histopathological features and biochemical markers of lung injury, reduce the expression and secretion of inflammatory cytokines, and effectively inhibit the fibrosis process [149]. Another study has shown that by enhancing the activity of the sarco/ER calcium ATPase 2a isoform (SERCA2a) through targeted gene therapy, the progression of PF can be effectively slowed down or reversed [150].

However, gene therapy for PF remains in the research stage and has not yet become a routine treatment method in clinical practice. Although preliminary research has achieved some positive results in the treatment of PF, its widespread clinical application still requires further realization.

#### 4.1.6. Immunomodulatory Therapy

Immune regulation is crucial in the development of PF [151]. Shenderov et al. highlighted the critical role of the immune system in PF and proposed the potential for developing immunomodulatory therapies that could effectively block or even reverse the condition [152]. This therapy aims to regulate the body's aberrant immune response, suppress inflammation, and halt the progression of fibrosis. Van den Bosch et al. noted that immunomodulatory therapy remains the cornerstone treatment strategy for PF, encompassing the use of therapeutic agents such as glucocorticoids, mycophenolate mofetil, azathioprine, cyclophosphamide, and rituximab [153].

From a clinical perspective, due to the close association between the pathological process of PF and the immune response, immunomodulatory therapy can be broadly applied as a treatment option for various types of PF patients. However, further research is essential to refine treatment protocols and assess the safety and efficacy of these interventions.

### 4.2. TCM-Related Compounds and Ingredients

Although pirfenidone and nintedanib, the main drugs used to treat PF, can delay disease progression, they cannot reverse PF. Moreover, they are expensive and cause side effects; thus, most patients are unable to take them for a long time. Numerous studies have shown that TCM is effective and safe for treating PF and has broad application prospects. An increasing number of recent studies have explored the effects and mechanisms of TCM prescriptions and active ingredients in the treatment of PF, which has gradually become a popular topic in PF research. A growing body of the literature in the field of TCM has also focused on identifying safer and more effective antifibrotic drugs.

At present, research on the prevention and treatment of PF with TCM is mainly focused on single TCM medicines and their extracts as well as TCM compound formulas. The main mechanisms and processes underlying the therapeutic effects of TCM in PF are the TGF-β1/Smads signaling pathway, the hedgehog signaling pathway, the PI3K/Akt signaling pathway, the NF-κB–snail pathway, the Wnt pathway, oxidative stress, autophagy, and EMT. In the following sections, we have compiled some information regarding the use of single TCM medicines and TCM formulas for the treatment of PF and described their mechanisms of action for clinical reference and application.

#### 4.2.1. Single TCM Medicines and Their Extracts

Single herbal medicines are compounds derived from TCM drugs that exhibit specific pharmacological activities. These components typically possess well-defined chemical structures and pharmacological effects that enable them to exert therapeutic benefits in particular diseases or pathological processes. An inflammatory response is commonly observed during the progression of PF. Single herbal medicines can mitigate this pulmonary inflammation by suppressing the release of inflammatory mediators and the infiltration of inflammatory cells, thereby decelerating the advancement of PF. PF is intricately linked to oxidative stress, and some single herbal medicines possess robust antioxidant properties.

These medicines can neutralize free radicals within the body and safeguard lung tissue from oxidative damage. Furthermore, single herbal medicines and their extracts can directly inhibit the proliferation of fibroblasts and the synthesis of collagen, thereby diminishing the development of PF. In addition, PF is associated with aberrant immune responses. The active constituents of some single herbal medicines can modulate the activity and function of immune cells, thereby restoring the equilibrium of the immune system. The current research findings are summarized in Table 1.

#### 4.2.2. TCM Prescriptions

TCM prescription therapy for PF is a treatment method based on TCM theory. It targets the complex pathogenesis of PF, relieves symptoms, improves lung function, and delays disease progression through the synergistic effects of multiple Chinese herbs.

Research on TCM formulas for treating PF mainly involves self-formulated and classic formulas, such as the Xuanfei Baidu decoction, Jiegeng decoction, Danggui Buxue Tang, and Yupingfeng San (Table 2). They mainly exert antifibrotic effects by regulating lung fibroblast differentiation, EMT, apoptosis, oxidative stress, profibrotic factors (such as TGF, CTGF, and VEGF), the PI3K/Akt signaling pathway, the JAK-STAT signaling pathway, and miRNAs.

#### 4.2.3. External Therapies in TCM

TCM theory includes an extensive body of experience for the external treatment of PF. External TCM treatment involves therapeutic practices conducted on the body's surface, which are guided by the principles of TCM theory. These methods do not involve the ingestion of oral medications but instead focus on stimulating the body's surface meridians and acupoints. They offer unique benefits in alleviating symptoms, enhancing the quality of life, and extending survival rates. The specific external TCM treatment modalities for PF primarily include acupuncture, moxibustion, cupping, and gua sha (scraping).

However, the experimental research on TCM treatment for PF exhibits certain limitations, including inconsistent animal modeling approaches, inadequate scientific research design, and a scarcity of multicenter, large-sample, and high-quality randomized controlled trial data. Future endeavors should prioritize enhancing the evidence-based medical foundation for TCM interventions in PF.

### 4.3. Nondrug Treatment

#### 4.3.1. Oxygen Therapy

Oxygen therapy can improve the patient's hypoxic condition. Although there is no direct evidence that oxygen therapy affects the prognosis of patients with PF and hypoxemia, indirect evidence from chronic obstructive pulmonary disease indicates that long-term oxygen therapy can significantly improve patient outcomes.

#### 4.3.2. Pulmonary Rehabilitation

Pulmonary rehabilitation is an intervention for patients with chronic lung disease who experience symptoms such as chest tightness and shortness of breath after exercise, as well as a reduced ability to perform daily activities. Its goal is to alleviate symptoms and slow the progression of the disease.

#### 4.3.3. Dietary Guidance

Individuals with lung disease may experience weight loss due to the discomfort of eating and the increased energy expenditure required for breathing. Nonetheless, a nutritionally dense diet that provides sufficient calories is crucial. Strive to consume smaller, more frequent meals throughout the day. Aim to incorporate a varied intake of fruits and vegetables, whole grains, low-fat or fat-free dairy, and lean meats into your diet. Avoid trans fats, saturated fats, excessive salt, and added sugars. A dietitian can offer additional guidance for maintaining a healthy diet.

#### 4.3.4. Get Vaccinated

Respiratory infections can worsen symptoms of PF. Thus, receiving vaccinations for pneumonia and influenza may be potential measures to prevent the acute exacerbation of PF [206, 207]. Additionally, during flu season, it is advisable to minimize visits to crowded places as much as possible.

In addition, PF can be associated with gastroesophageal reflux disease, pulmonary arterial hypertension, and lung cancer. When combined with pulmonary arterial hypertension, it can severely impact the function of the lungs and heart, resulting in symptoms such as difficulty breathing and fatigue, and a poor prognosis. However, when PF is combined with lung cancer, the prognosis is often extremely poor. The incidence of lung cancer in patients with PF is much higher than in those without, and the condition progresses rapidly. Therefore, for such patients, it is necessary to develop personalized treatment plans and actively monitor changes in the condition to prolong survival and improve quality of life.

## 5. Conclusions

The mechanisms underlying PF are complex and include damage to AECs, aggregation and activation of inflammatory cells and chemotactic factors, fibroblast proliferation, myofibroblast transformation, production and deposition of large amounts of collagen, autophagy, EMT, and misregulation of mitochondrial quality control, miRNAs, and circRNAs. In terms of treatment, none of the drugs used in modern medicine can significantly improve PF, and lung transplantation is the only curative option at present. However, these medications can inhibit PF progression to some extent and improve lung function. The representative drugs are pirfenidone and nintedanib, which are relatively safe for clinical applications. Depending on the specific condition, if a patient has excessive phlegm and gastric acid reflux disease, these drugs can be combined with NAC or antacids.

The research on the use of TCM for the prevention and treatment of PF has primarily focused on single TCM medicines and their extracts and TCM prescriptions. The main pathways and mechanisms involved in the effects of these drugs include the TGF-β1/Smads signaling pathway, the hedgehog signaling pathway, the PI3K/Akt signaling pathway, the NF-κB-Snail pathway, the Wnt signaling pathway, oxidative stress, autophagy, EMT, and RNAs. These signaling pathways are not independent but are interconnected and influenced by each other.

Chinese medicine monomers, Chinese medicine compounds, and their active ingredients can effectively improve the symptoms of fibrosis in experimental animals and delay disease progression, showing good anti-inflammatory, antioxidant, and antifibrotic effects.

These results indicate that TCM has good clinical prospects for preventing and treating PF, especially in prescriptions with the characteristics and advantages of TCM. However, the composition of TCM formulas is complex, and their mechanisms require elucidation through a combination of network pharmacology, artificial intelligence, and in vivo and in vitro experiments. Further research should be conducted on the interventions and treatments for PF, the identification of effective active ingredients, and the development of internationally recognized and clinically proven antifibrotic drugs.

## Figures and Tables

**Table 1:** Single traditional Chinese medicine and its extracts.

Cellular targets and pathways	Name of drug	Action on targets and pathways	Reference
TGF-β/Smads signaling pathway	Total flavonoids of *Oxytropis falcata* Bunge	Regulation of the TGF-β1/Smads signaling pathway by significantly reducing the expression of TGF-β1, p-Smad2, and p-Smad3, and increasing the expression of Smad7.	[154]
	*Arenaria kansuensis*	Activation of the Nrf2 pathway and inhibition of the NF-κB/TGF-β1/Smad2/3 pathway.	[155]
	Polydatin	By suppressing the TGF-β/Smad signaling pathway, it inhibits inflammation and EMT; by inhibiting IL-1β, TNF-α, oxidative stress biomarker MDA, α-SMA, and hydroxyproline, it exerts anti-inflammatory, antioxidant, and antifibrotic effects.	[156, 157]
	Baicalein	Downregulation of miR-21 to inhibit TGF-β1–induced lung fibroblast differentiation.	[158]
	Tanshinone IIA	Inhibition of Smads phosphorylation induced by TGF-β1, especially Smad3 in nucleus, and upregulation of Smad7 expression, resulting in decreased ECM deposition.	[159, 160]
	Salvianolic acid B	Suppression of TGF-β-induced myofibroblastic differentiation of MRC-5 fibroblasts and TGF-β-mediated EMT by inhibiting both Smad-dependent signaling and the Smad-independent MAPK pathway.	[161]
	Glycyrrhizic acid	Ameliorating BLM-induced pulmonary fibrosis and activation of TGF-β signaling pathway in the lungs.	[162]
	Andrographolide	By adjusting the TGF-β1/Smad2/3 or TGF-β1/ERK1/2 pathway, it inhibits fibroblast proliferation and differentiation.	[163]
	Curcumin	Inhibiting the expression of TGF-β regulates the process of EMT in PF, while reducing the activity of MMP-9 and the expression of α-SMA, TIMP-1, and CTGF.	[164, 165]
	Magnolol	Inhibition of hydroxyproline (HYP), MPO activity, and TNF-α and TGF-β levels in lung tissue, and significant enhancement of SOD activity, thereby inhibiting excessive collagen deposition.	[166]
	Safflor yellow	Inhibition of the proliferation of α-SMA-positive cells and the expression of TGF-β1 in PF rats, thereby reducing HYP levels.	[167]
	Paclitaxel	Suppression of the TGF-β1/Smad3 signaling pathway and upregulation of miR-140 levels can reverse the EMT process in lung epithelial cells.	[168]
	Schisandrol A	Adjusting the TGF-β signaling pathway.	[169]
	*Nervilia fordii* extract	Downregulation of the protein and RNA expression of TGF-β1, α-SMA, Smad3/4, p-Smad3/4, CTGF, and p-ERK1/2, and upregulation of Smad7 and ERK1/2, thereby reducing inflammation, oxidation, and collagen deposition.	[170]
CTGF	Baicalein	Downregulation of CTGF to alleviate the production of Col I in lung tissue.	[171]
EMT	Triptolide	Inhibiting TGF-β1-mediated EMT; reducing IL-1β and IL-13.	[172]
	Osthole	By inhibiting the NF-κB-Snail pathway, it reverses EMT process induced by TGF-β1.	[173]
	Paeoniflorin	Reducing snail protein expression by upregulating Smad7, thereby inhibiting TGF-β-induced EMT.	[174]
	Salidroside	Inhibition of IκBα phosphorylation and NF-κB p65 nuclear accumulation; downregulation of vimentin, fibronectin, α-SMA, TGF-β1, and p-Smad2/3, upregulation of the expression of E-cadherin and activation of the Nrf2 antioxidant signaling pathway to reverse TGF-β1–induced EMT of alveolar epithelial cells.	[175]
	Total flavonoid extract from *Dracocephalum moldavica* L.	Interfering with the hedgehog signaling pathway by reducing the expression of Shh, Ptch1, and SMO proteins, thereby inhibiting the production of downstream target gene Gli1 and significantly reducing the expression of Col I, fibronectin, and α-SMA.	[176]
	Emodin	Alleviating PF and EMT by regulating the c-MYC/miR-182-5p/ZEB2 axis.	[177]
Autophagy	Ligustrazin	Improving autophagy levels and miR-193a expression in lung cells, inhibiting activation of the PI3K/Akt/mTOR and hedgehog signaling pathways, alleviating oxidative damage, and inhibiting expression of VEGF and fibrinogen.	[178]
	Isoliquiritigenin	Activating autophagy in lung fibroblasts via suppressing the PI3K/AKT/mTOR pathway, and decreasing the expression of α-SMA, type I collagen, and fibronectin.	[179]
	Ellagic acid	Inhibiting the Wnt signaling pathway, promoting autophagy and apoptosis of myofibroblasts, and inhibiting fibroblast activation and extracellular matrix production.	[180]
	Oxymatrine	Activating the PI3K/Akt signaling pathway to weaken TGF-β1–mediated mitochondrial apoptosis in alveolar epithelial cells.	[181]
	Atractylenolide III	Suppressing autophagy by mediating mTOR-dependent signaling pathways in alveolar macrophages (AM) and reducing AM apoptosis.	[182]
	Berberine	Upregulation of PTEN to inhibit the PI3K/Akt signal transduction pathway, targeted inhibition of P-mTOR activation, and promotion of the expression of ATG6 homolog (Beclin 1), LC3-II, and the formation of autophagosomes.	[183]
	Resveratrol	Activation of autophagy, prevention of mitochondrial dysfunction, promotion of the clearance of pathological ROS, and reduction of oxidative damage; Inhibiting the activation of NLRP3 inflammasomes inhibits autophagy of AM.	[184, 185]
	Quercetin	Preventing lipopolysaccharide-induced decreases in ATG5 and LC3, thereby increasing autophagy activity and significantly downregulating the expression of profibrotic factors TGF-β1, VEGF, and IL-6.	[186]
	Celastrol	Upregulating the expression of Beclin1 and Vps 34, promoting the formation of ATG5-Atg12-Atg16, inducing lipoylation of LC3-I to LC3-II, and thereby promoting the generation of autophagosomes.	[187]
	Dioscin	Promoting the upregulation of LC3-II and Beclin1, as well as the downregulation of p62, enhancing the autophagy level of AM to inhibit pathological ROS production, thereby reducing the activation of mitochondrial-dependent apoptosis pathways.	[188]
	Astragalin	Inhibition of the expression of autophagy-related proteins beclin-1 and LC3A/B, as well as downregulation of E-cadherin and upregulation of vimentin expression in lung epithelial cells.	[189]
	Puerarin	Alleviating silicon dioxide-induced pulmonary inflammation and fibrosis via improving autophagolysosomal dysfunction.	[190]
NF-κB-Snail pathway	Ginsenoside Rb1	Suppressing NLRP3 inflammasome activation and the NF-κB pathway.	[191]

Abbreviations: α-SMA = α-smooth muscle actin, AM = alveolar macrophages, ATG = autophagy-related genes, CTGF = connective tissue growth factor, ECM = extracellular matrix, EMT = epithelial–mesenchymal transition, ER = endoplasmic reticulum, HYP = hydroxyproline, IPF = idiopathic pulmonary fibrosis, MDA = malondialdehyde, PF = pulmonary fibrosis, ROS = reactive oxygen species, SM = sulfur mustard, SMO = smoothened, TGF-β1 = transforming growth factor-β1, and VEGF = vascular endothelial growth factor.

**Table 2:** Prevention and treatment of PF with TCM decoction.

Cellular targets and pathways	Name of drug	Action on targets and pathways	Reference
IL-6/STAT3 signaling pathway	Xuanfei Baidu decoction	Inhibition of collagen deposition by fibroblasts in the lungs; downregulation of α-SMA level, which inhibits fibroblast migration; inhibition of IL-6/STAT3 activation and infiltration of related macrophages.	[192]
PI3K/Akt signaling pathway	Jiegeng decoction	Regulation of the expression of apoptosis-related proteins such as Bax, Caspase3, Caspase8, Caspase9, and Bcl-2 through the PI3K/Akt signaling pathway, and inhibition of type-II AECs apoptosis.	[193]
	Yifei Sanjie formula	Inhibiting the PI3K/Akt/mTOR signaling pathway, activating autophagy, and reducing expression of α-SMA, Col I, and Col III, inhibiting abnormal collagen accumulation.	[194]
TGF-β/Smads signaling pathway	Xin Jia Xuan Bai Cheng Qi decoction	Inhibition of Smad2 and p-Smad2 activity induced by TGF-β1, promotion of Smad7 expression and decrease of α-SMA and fibronectin activity.	[195]
	Danggui Buxue Tang	Suppression of the TGF-β1/Smad3/PAI-1 signaling pathway and the TLR4/NLRP3 signaling pathway; improving PF through microRNA and mRNA regulatory network	[196–198]
Senescence	Yiqi Huayu decoction	Reducing the aging-related markers such as p53, p21, and p16; inhibiting the production of ROS in lung tissue; and reducing the expression of SASP in serum.	[199]
EMT	Total extract of Yupingfeng	Inhibiting EMT process, reducing HMGB1 activity and TGF- β1 activation.	[200, 201]
CTGF	Buyang Huanwu decoction	Downregulation of CTGF and AKT mRNA levels, reduction of serum Col I and Col III levels, and reduction of CTGF and p-AKT protein expression; inhibiting the PI3K/Akt signaling pathway to suppress TGF-β–induced collagen deposition and EMT.	[202, 203]
VEGF	Yangfei Huoxue decoction	Inhibition of the expression of vascularized VEGF and IL-1β.	[204]
JAK-STAT signaling pathway	Feifukang	Inhibition of the JAK–STAT signaling pathway.	[205]

Abbreviations: AEC = alveolar epithelial cell, CTGF = connective tissue growth factor, ROS = reactive oxygen species, SASP = secretory phenotype, SMA = smoothened, TGF-β1 = transforming growth factor-β1, and VEGF = vascular endothelial growth factor.

## Data Availability

The authors have nothing to report.

## Nomenclature

α-SMA: α-Smooth muscle actin
AEC: Alveolar epithelial cell
AM: Alveolar macrophages
ATG: Autophagy-related genes
ATP: Adenosine triphosphate
CTD: Connective tissue disease
CTGF: Connective tissue growth factor
DUBs: Deubiquitination enzymes
ECM: Extracellular matrix
EMT: Epithelial–mesenchymal transition
ER: Endoplasmic reticulum
FDA: Food and Drug Administration
FF: Fibroblast foci
FLT: Fms-like tyrosine kinase
FVC: Forced vital capacity
GGO: Ground-glass opacity
GRP: Glucose regulatory protein
HYP: Hydroxyproline
HRCT: High-resolution computed tomography
IPF: Idiopathic pulmonary fibrosis
MDA: Malondialdehyde
MMP: Matrix metalloproteinases
MSC: Mesenchymal stem cell
mTOR: Mammalian target of rapamycin
mtROS: Mitochondrial ROS
nRTKs: Nonreceptor tyrosine kinases
PDGF: Platelet-derived growth factor
PDGFR: Platelet-derived growth factor receptor
PF: Pulmonary fibrosis
PGC: Peroxisome proliferator-activated receptor-γ coactivator
PTPN13: Protein tyrosine phosphatase N13
RAS: Restrictive allograft syndrome
RBP: RNA-binding proteins
ROS: Reactive oxygen species
RTK: Receptor tyrosine kinases
SA-β-gal: Senescence-associated β-galactosidase
SASP: Secretory phenotype
SERCA2: Asarco/endoplasmic reticulum calcium ATPase 2a isoform
SLB: Surgical lung biopsy
SM: Sulfur mustard
SMO: Smoothened
SPP: Secretory pyrophosphoprotein
STAT: Signal transducer and activator of transcription
TBLC: Transbronchial lung cryobiopsy
TCM: Traditional Chinese medicine
TGF-β1: Transforming growth factor-β1
TIMP: Tissue inhibitor of matrix metalloproteinase
UIP: Usual interstitial pneumonia
VEGF: Vascular endothelial growth factor
VEGFR: Vascular endothelial growth factor receptor.
